# The Role of Vitamin D Levels in Optimizing Treatment for Pediatric Inflammatory Bowel Disease (IBD) Patients and an Examination Into Different Factors That Influence IBD Treatment Outcomes

**DOI:** 10.7759/cureus.68055

**Published:** 2024-08-28

**Authors:** Safia Centner, Chelsea Wu, Thinzar Zaw, Pablo Palomo, Hadeel A Haddad

**Affiliations:** 1 Medicine, University of Central Florida College of Medicine, Orlando, USA; 2 Pediatric Gastroenterology, Nemours Children’s Hospital, Orlando, USA

**Keywords:** vitamin d, pediatric crohn’s disease, fecal calprotectin, ulcerative colitis (uc), inflammatory bowel disease

## Abstract

Background

Inflammatory bowel disease (IBD), including Crohn’s disease (CD) and ulcerative colitis (UC), presents significant challenges, particularly in pediatric patients. Vitamin D deficiency has been associated with IBD, but its role in disease activity and remission remains unclear. This study investigates the relationship between serum vitamin D levels and IBD markers, including Pediatric Crohn’s Disease Activity Index (PCDAI), Pediatric Ulcerative Colitis Activity Index (PUCAI), fecal calprotectin levels, and endoscopy findings. It also explores racial and ethnic disparities in these relationships.

Methodology

A retrospective study was conducted involving 51 pediatric patients with IBD from the Nemours Children’s Health EMR system. Inclusion criteria required documented serum vitamin D levels at diagnosis and post-treatment, and at least one post-treatment assessment of PUCAI/PCDAI, calprotectin, or endoscopy. The study employed Spearman and Pearson correlation tests to analyze the associations between vitamin D levels and IBD markers. Ethnicity and race were analyzed using t-tests and chi-square tests.

Results

No statistically significant correlations were found between changes in serum vitamin D levels and IBD markers (endoscopy results, calprotectin levels, PUCAI/PCDAI scores) for both UC and CD. Analysis of racial and ethnic disparities revealed that Hispanic patients had significantly higher post-treatment calprotectin levels compared to non-Hispanics, although other markers showed no significant differences. Vitamin D levels did not significantly differ between racial or ethnic groups.

Conclusions

This study found no significant correlation between serum vitamin D levels and IBD activity markers in pediatric patients. Despite initial hypotheses, vitamin D levels do not appear to be useful in assessing IBD remission or disease state. Racial and ethnic disparities in IBD severity were observed, but further research with larger sample sizes and more consistent data collection is needed to draw more definitive conclusions.

## Introduction

Inflammatory bowel disease (IBD) is an autoimmune inflammatory condition that affects the gastrointestinal tract. It primarily includes two major disorders: ulcerative colitis (UC) and Crohn’s disease (CD). UC is confined to the colon and rectum, presenting with continuous mucosal inflammation, while CD can affect any part of the gastrointestinal tract, from the mouth to the anus, and is characterized by transmural inflammation and skipped lesions [1,2]. Both conditions can significantly impact a patient’s quality of life, particularly in the pediatric population who face unique challenges due to their ongoing growth and development [3]. Symptoms of IBD include, but are not limited to, abdominal pain, abnormal bowel movements, bloating, weight loss, and blood in the stool [4].

Among the various complications and comorbidities associated with IBD, vitamin D deficiency has garnered considerable attention [4]. Both CD and UC are associated with lower vitamin D levels; however, whether or not IBD is a causative agent for the lower vitamin D levels seen in pediatric IBD patients or if vitamin D deficiencies increase the risk of subsequently developing IBD is still unclear [5,6]. Vitamin D is essential to the maintenance of normal cellular and body function due to its anti-inflammatory, immune, and regulatory properties [7]. It is thought to modulate inflammation through moderating the production of cytokines and immune cells, which help serve as our body’s defense mechanism [8].

Some research has suggested that low serum vitamin D levels correlate with increased disease activity and poorer outcomes in IBD patients [9]. In comparing a control group with an experimental group that had been provided with vitamin D supplements, results showed that those with vitamin D had a markedly lower IBD activity score and a significantly higher quality of life score. Along with these changes in scores, levels of several inflammatory markers decreased as well. This study concluded that vitamin D had a significantly inverse relationship with IBD activity and frequency of hospitalization due to IBD [1].

To determine the amount of inflammation present within the gastrointestinal tract, three measurement tools can be used, namely, biomarkers (such as fecal calprotectin), endoscopies/colonoscopies, and Pediatric Crohn’s Disease Activity Index (PCDAI)/Pediatric Ulcerative Colitis Activity Index (PUCAI). The PCDAI, completed by physicians, includes symptoms, physical examination, and growth and inflammatory markers and is scored from 0-100, with higher scores indicative of a more severe case. The PUCAI is similar in what it is measuring, with higher scores suggesting a more serious, severe case [10]. Calprotectin levels are also used to assess current inflammation in a patient. It is a biomarker that appears in feces if patients have intestinal inflammation [11]. All three provide information about IBD severity and activity in patients; however, there is limited literature on the possible association between patient vitamin D levels and these markers.

Regarding the relationship between vitamin D and specific IBD diagnosis, a study of 403 patients with CD and 101 with UC indicated a relationship between vitamin D levels and disease activity in those with CD, but not with UC [12]. Another study with 3,217 participants, 1,769 with CD, found that lower vitamin D levels in CD patients were associated with increased surgeries and hospitalizations than those with higher levels. A few other studies also indicated this relationship, whereas another study showed no correlation between CD and vitamin D levels. This data is lacking in patients with UC, requiring further evaluation [12].

Race and ethnicity are critical factors that influence the incidence, prevalence, and clinical outcomes of IBD. Studies have shown that IBD incidence varies significantly among different racial and ethnic groups, with increasing rates observed among minority populations in recent years [13]. For instance, African American and Hispanic children have been reported to present with more severe disease phenotypes and are more likely to have extensive disease at diagnosis compared to their Caucasian counterparts [14]. Vitamin D deficiency has been identified as more prevalent among certain racial and ethnic groups, which may contribute to the observed differences in IBD severity and outcomes. This deficiency can exacerbate the inflammatory processes in IBD, leading to more severe disease presentations and complications [15]. Further research is needed to explore the biological, environmental, and socioeconomic factors contributing to these disparities and to develop targeted interventions that can mitigate their impact.

Despite the recognized importance of vitamin D in IBD, gaps remain in the literature regarding the relationship between serum vitamin D levels and IBD severity, particularly in pediatric populations. Moreover, the potential of vitamin D treatment to predict or influence IBD treatment outcomes remains underexplored. To address these gaps, this collaborative study aims to explore the following three objectives: (1) to investigate the possible association between post-treatment vitamin D levels in pediatric patients with IBD and subsequent markers of IBD remission; (2) to investigate possible differences between serum vitamin D levels, calprotectin stool levels, PUCAI/PCDAI scores, and endoscopy results between pediatric UC and CD patients; and (3) to compare those same variables between pediatric patients of different races. The results of this investigation may provide insights into approaches to optimize the treatment and care of pediatric IBD patients and how serum vitamin D levels may provide a less invasive way to assess IBD remission.

## Materials and methods

This study is a retrospective study involving 51 patients identified from Nemours Children’s Health EMR system, EPIC.

Inclusion and exclusion criteria

We included the following patients: (1) those aged 0-18 years; (2) those with a clinical diagnosis of IBD (either CD or UC); (3) those with documented serum vitamin D 25-Hydroxy levels at both the time of IBD diagnosis and following IBD treatment; and (4) at least one documented post-treatment PUCAI/PCDAI score, calprotectin stool level, or colonoscopy procedure done at Nemours Children’s Health.

We excluded the following patients: (1) those aged over 18 years; (2) those with no clinical diagnosis of IBD (either CD or UC); and (3) those with no documented serum vitamin D 25-Hydroxy levels at both the time of IBD diagnosis and following IBD treatment.

The values at the time of diagnosis had to be within two months of diagnosis, and post-treatment values had to be at least seven months after the date of diagnosis. Because the endoscopies and colonoscopies were reported qualitatively, for statistical analysis purposes, the results were given a numerical score, ranked from 0 to 5 based on the severity of the findings.

Data analysis

SPSS software (IBM Corp., Armonk, NY, USA) was used for statistical testing.

Part 1: The Role of Vitamin D Levels in Evaluating Treatment for Pediatric Inflammatory Bowel Disease Patients

A two-tailed Pearson correlation test was used for continuous variables (PUCAI/PCDAI scores, calprotectin levels), and a two-tailed Spearman’s correlation test was used for ordinal variables (colonoscopy results). Colonoscopy results were graded on a scale of 0-5 based on qualitative findings (0 = no IBD findings, 5 = severe IBD findings). For this study, we considered a marker to be “post-treatment” if it was recorded at least seven months from the diagnosis date.

Part 2: Comparing Patients With Ulcerative Colitis and Those With Crohn’s Disease to Determine if Vitamin D Levels Can Be Used as an Inflammation Marker for Inflammatory Bowel Disease Status and if It Is More Specific for One Over the Other

The changes/differences between each respective post-treatment and values at diagnosis were calculated by subtracting the value at the time of diagnosis from the post-treatment to determine if there was an overall increase or decrease. Two-tailed Spearman correlation tests were used to assess the correlation between changes in vitamin D levels and changes in procedure scores for both UC (N = 9) and CD (N = 23). Two-tailed Pearson correlation tests, performed through SPSS, were used between changes in vitamin D levels and changes in calprotectin levels and PUCAI/PCDAI scores for both UC. Eight patients with UC had data for changes in calprotectin levels, and three with UC had data for the PUCAI scores. For CD, 33 patients had data for calprotectin levels, and one had data for PCDAI. Because of the limited data for CD and PCDAI, the correlation could not be calculated.

Part 3: Racial and Ethnic Disparities in Vitamin D Levels and Remission Markers Among Pediatric Inflammatory Bowel Disease Patients

Race (Caucasian and other) and ethnicity (non-Hispanic and Hispanic) were reclassified into a binary categorical variable due to the lack of participants and representation of minority groups. Independent sample t-tests were used for continuous variables (calprotectin and vitamin D levels) and analyzed for significance with normality tests, i.e., Welch’s t-test and Mann-Whitney U test, respectively. Colonoscopy results were graded on a scale of 0-5 based on qualitative findings. This was recategorized into -1 (improved), 0 (remained same), and 1 (worsened) depending on pre- and post-treatment grades. The chi-square test was used for colonoscopy change.

## Results

Part 1: The role of vitamin D levels in evaluating treatment for pediatric inflammatory bowel disease patients

**Figure 1:**
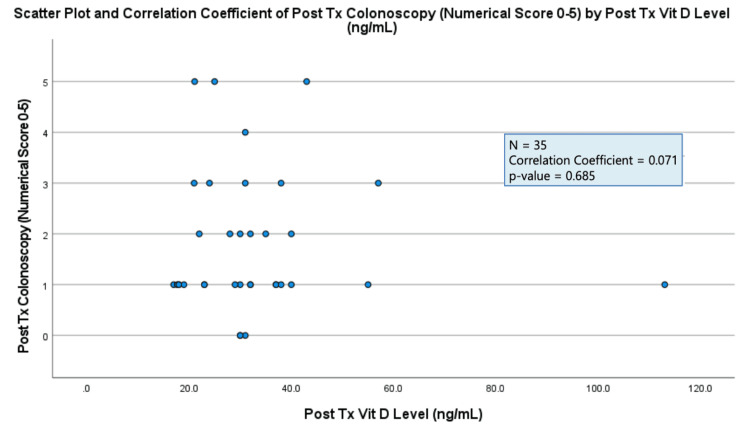
Plot demonstrating two-tailed Spearman correlation test between post-treatment (Tx) colonoscopy results and post-treatment (Tx) serum vitamin D levels.

**Figure 2:**
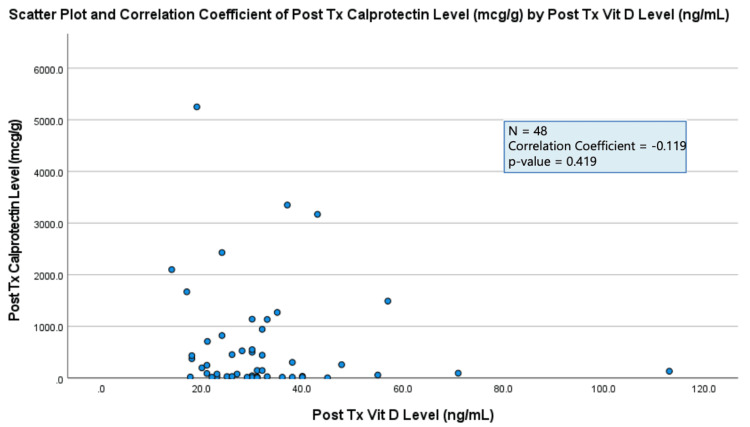
Plot demonstrating two-tailed Pearson correlation test between post-treatment (Tx) stool calprotectin levels and post-treatment (Tx) serum vitamin D levels.

**Figure 3:**
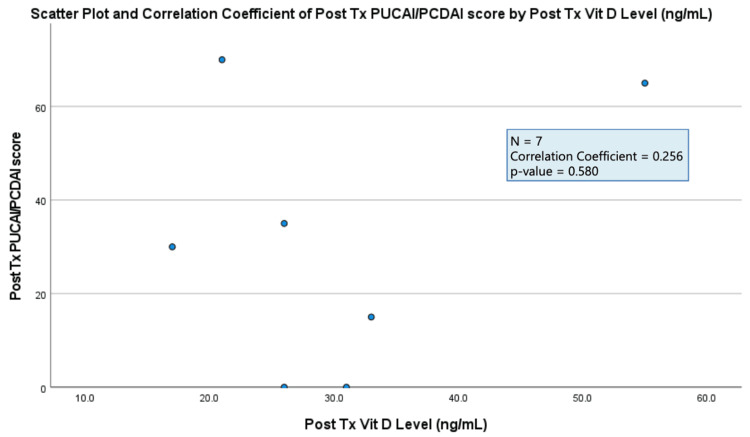
Plot demonstrating two-tailed Pearson correlation test between post-treatment (Tx) Pediatric Ulcerative Colitis Activity Index (PUCAI)/Pediatric Crohn’s Disease Activity Index (PCDAI) scores and post-treatment (Tx) serum vitamin D levels.

The results of each data analysis showed a negligible correlation between all three markers of IBD activity (colonoscopy results, stool calprotectin level, PUCAI/PCDAI score) and serum vitamin D levels (correlation coefficient = 0.00-0.30) (Figures 1-3). All three results were found to not be statistically significant (p-value > 0.05) (Figures 1-3).

Part 2: Comparing patients with ulcerative colitis and those with Crohn’s disease to determine if vitamin D levels can be used as an inflammation marker for inflammatory bowel disease status and if it is more specific for one over the other

**Figure 4:**
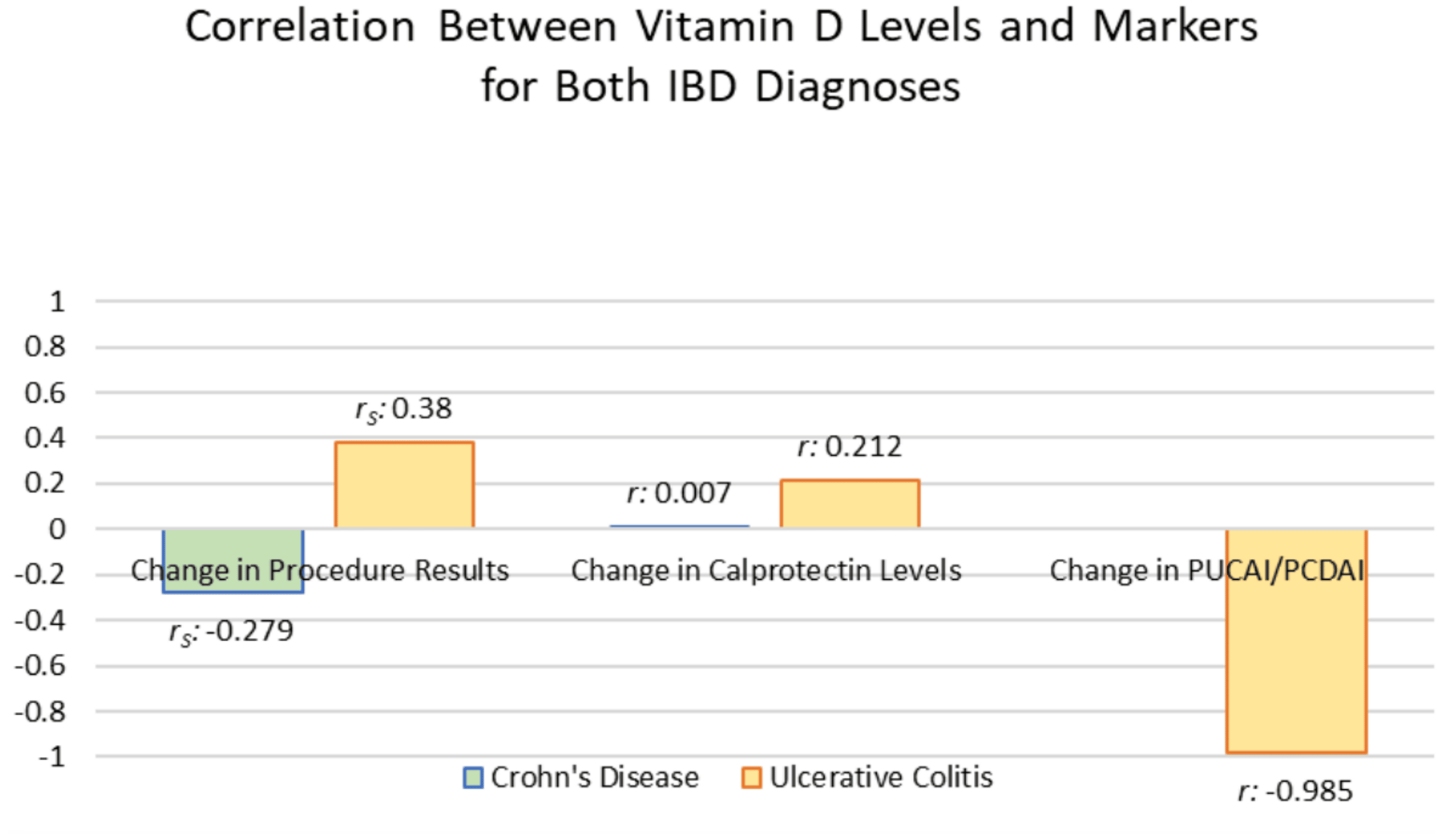
Correlation between change in vitamin D Levels and inflammatory bowel disease (IBD) markers between ulcerative colitis and Crohn’s disease, r_s_ = Spearman correlation; r = Pearson correlation.

**Figure 5:**
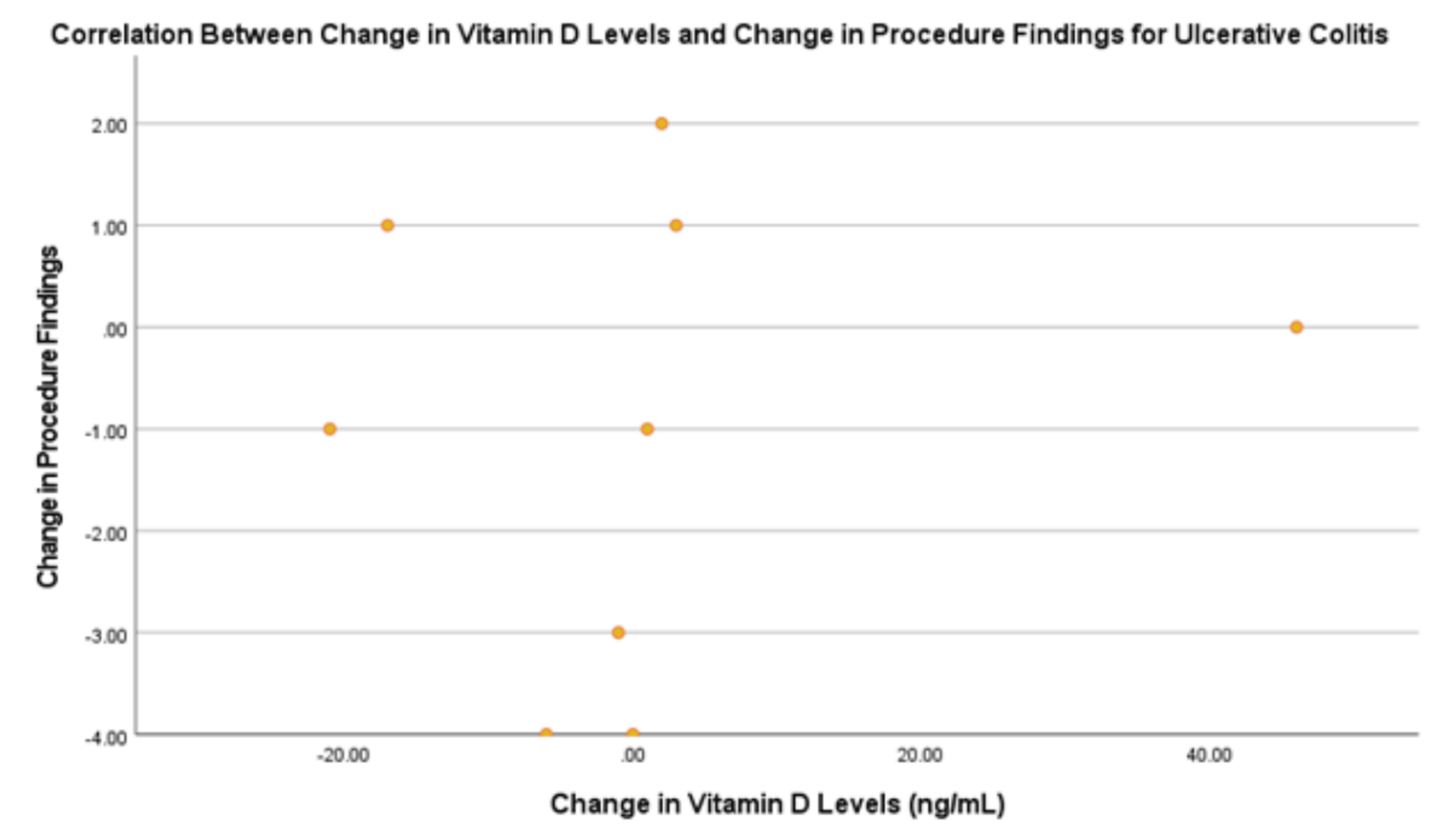
Correlation between change in vitamin D levels and procedure results for ulcerative colitis.

**Figure 6:**
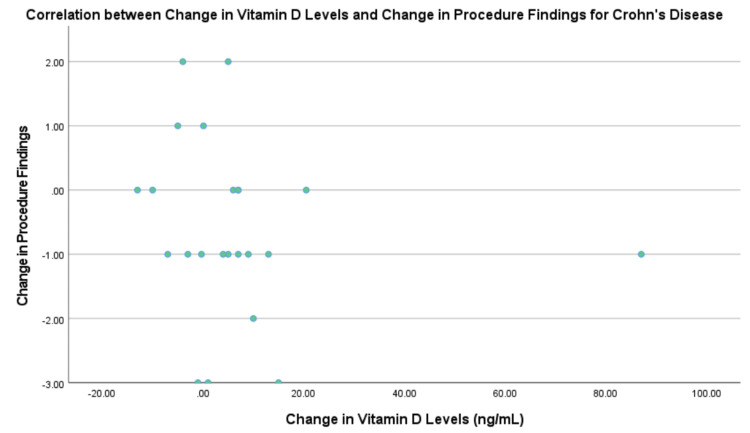
Correlation between change in vitamin D levels and procedure results for Crohn’s disease.

Figure 4 compares the correlation coefficients for both diseases with the respective IBD markers. The correlation between change in vitamin D levels and change in procedure results was not statistically significant for both UC and CD. For UC, there was a low positive correlation of 0.38 and significance of 0.313 (Figure 5). For CD, there was a negligible correlation of -0.279, with a significance of 0.197 (Figure 6). The procedure results were graphed due to the amount of data available for this marker.

The correlation between change in vitamin D levels and change in calprotectin levels was not statistically significant for either diagnosis. For UC, there was a negligible correlation of 0.212, with a significance of 0.615. For CD, there was a negligible correlation of 0.007, with a significance of 0.967.

The correlation of change in vitamin D levels with pediatric IBD index levels proved to be very high and negative (r = -0.985) for UC. This value was not statistically significant (p = 0.109). The correlation for CD could not be determined due to the limited data.

Part 3: Racial and ethnic disparities in vitamin D levels and remission markers among pediatric inflammatory bowel disease patients

**Figure 7:**
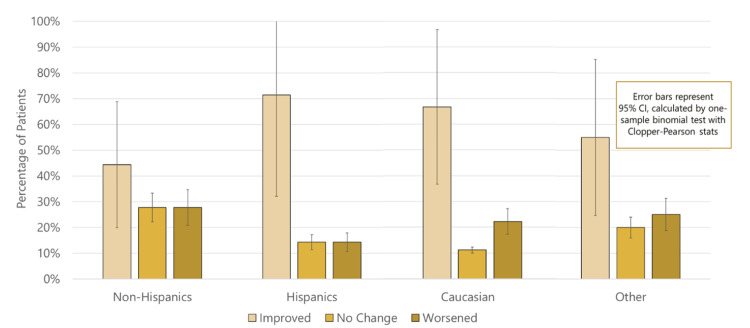
Comparison of changes in pre- and post-treatment colonoscopy grading by ethnicity and race.

**Figure 8:**
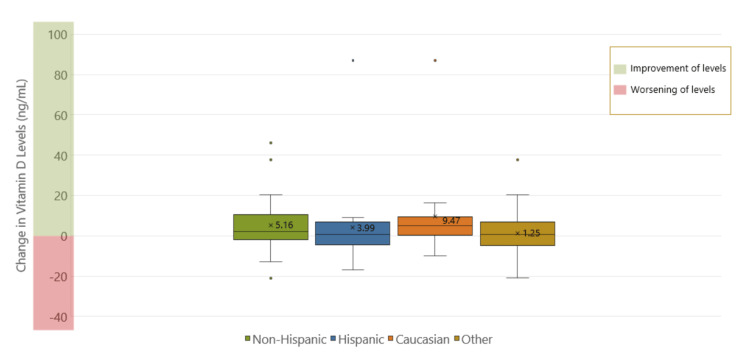
Comparison of changes in pre- and post-treatment vitamin D levels by ethnicity and race. There was no significant difference between pre- and post-treatment colonoscopy grading or vitamin D levels in either race or ethnicity.

**Figure 9:**
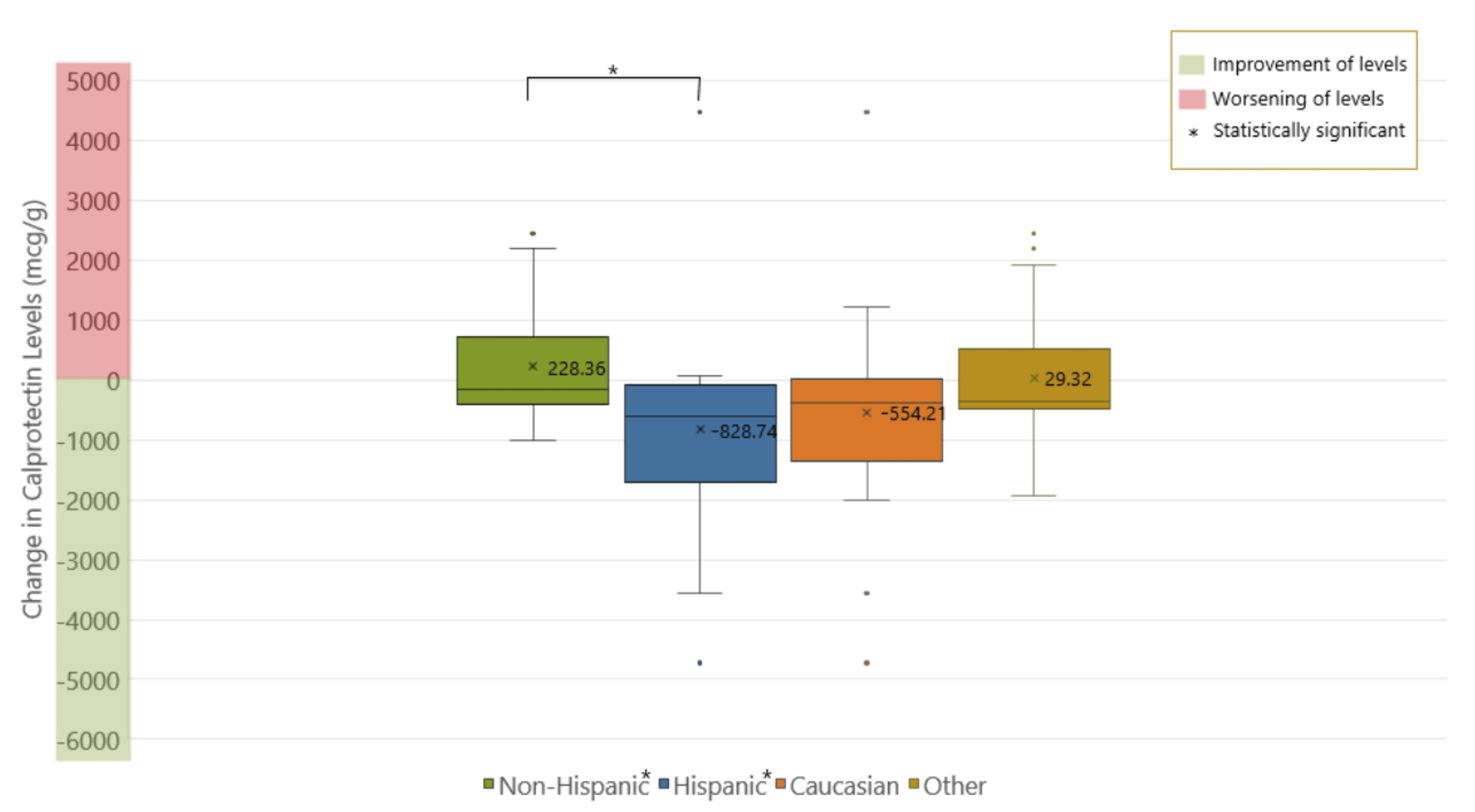
Comparison of changes in pre- and post-treatment calprotectin levels by ethnicity and race. Hispanics were found to have statistically significant higher post-treatment stool calprotectin levels (p = 0.01) compared to non-Hispanics (p < 0.05).

There was also no significance between pre- and post-treatment colonoscopy grading or vitamin D levels in either race or ethnicity (Figures 7, 8). Hispanics were found to have statistically significant higher post-treatment stool calprotectin levels (p = 0.01) compared to non-Hispanics (p < 0.05) (Figure 9).

## Discussion

IBD encompasses two primary disorders: UC and CD. Both conditions, though different in their characterizations, pose considerable challenges for pediatric patients, and thus, regular monitoring and check-ups are key to improving and maintaining disease stability [1-3]. Recent research has highlighted the potential role of vitamin D in modulating inflammation and immune responses in IBD, and, as a result, deficiency of this vitamin has been an area of focus [8]. The exact relationship remains unclear, especially in pediatric patients.

Part 1: The role of vitamin D levels in evaluating treatment for pediatric inflammatory bowel disease patients

Vitamin D levels were not found to be associated with disease remission when comparing them to classic markers of IBD disease activity. While research has shown that low serum vitamin D levels correlate with increased disease activity, this could not be determined based on the findings of this study [9]. This finding suggests that vitamin D levels may not be a reliable standalone indicator of IBD activity or severity. While this diverges from some studies that suggest a more pronounced relationship between vitamin D levels and IBD activity, the discrepancy may be attributed to differences in study design, sample sizes, and measurement methodologies. Additionally, the small sample size in this study likely limited the ability to detect subtle but clinically significant correlations.

Part 2: Comparing patients with ulcerative colitis and those with Crohn’s disease to determine if vitamin D levels can be used as an inflammation marker for inflammatory bowel disease status and if it is more specific for one over the other

Prior research has indicated that vitamin D levels may be more associated with disease activity in CD but not UC [12]. In this study, there was no difference between the two IBD diagnoses. For UC, there was a low positive correlation between changes in vitamin D levels and changes in PUCAI scores, although it was not statistically significant. The correlation between UC and PUCAI, though not significant, was very high, and the likely reason for this is the minimal number of patients who had a pediatric IBD index score. In contrast, CD showed negligible correlations across all markers. The limited data for CD, particularly regarding the PCDAI, highlights the need for larger, more comprehensive studies to fully understand these relationships.

Part 3: Racial and ethnic disparities in vitamin D levels and remission markers among pediatric inflammatory bowel disease patients

This study found that Hispanics had statistically significant higher post-treatment stool calprotectin levels compared to non-Hispanics, suggesting that they may have increased disease activity, despite treatment. This aligns with studies that have reported Hispanic children may present with worse disease phenotypes at baseline, thus influencing their response to treatment compared to those with less disease activity [14]. The higher calprotectin levels in Hispanic patients could indicate more severe or active disease, or it could reflect differences in vitamin D metabolism or absorption. Factors such as socioeconomic status, access to healthcare, and environmental conditions may also play a role in these outcomes.

Limitations

This study has several limitations. The sample size was small and with low power as it was unable to meet the previously anticipated sample size of 193 patients for 80% power. Additionally, there were issues with obtaining information from the EMR as patients’ ethnicity and race input appeared inaccurate and there were limited PUCAI/PCDAI scores available. More patients could help increase the validity of the results. Another limitation is that some patients only had one outcome variable to serve as a comparison. This one variable could have been affected for other reasons, so it would have been beneficial to have values for the other markers of inflammation. It is also difficult to compare variables as there were inconsistent timelines regarding the timing of the values. Some patients may have had data after only being treated for a year, whereas others might have had data after years of treatment. Ensuring that most, if not all, patients have regular comparison variable check-ups could help alleviate this issue.

There was a risk of confounding variables, especially as lax exclusion criteria were used. For example, other diseases or illnesses may have influenced the findings as patients with other non-IBD diagnoses were not excluded. There is a lack of representativeness as only Floridian patients were included which may have impacted the generalizability of the results to other populations not included in the study.

Further, retrospective studies can only show association, not causation, and can be affected by recall bias and differences in documentation or treatment.

## Conclusions

Although initially promising, based on the results of this investigation, serum Vitamin D levels do not seem to have any correlation with post-treatment colonoscopy results, stool calprotectin levels, or PUCAI/PCDAI scores and therefore cannot be used to assess IBD remission or disease state in pediatric patients. All correlations between vitamin D levels and the comparison markers for both UC and CD were also found to be not statistically significant and no difference between diagnoses could be ascertained.

There is weak evidence to suggest populations self-identifying as Hispanic are associated with lower remission proportions compared to non-Hispanics, as evidenced by the significantly higher post-treatment calprotectin levels. However, given the limitations of this study and the non-significance in the other remission variables, a clear association could not be reported.
